# Effect of biomass fuel use and kitchen location on maternal report of birth size: Cross-sectional analysis of 2016 Ethiopian Demographic Health Survey data

**DOI:** 10.1016/j.puhip.2021.100211

**Published:** 2021-10-29

**Authors:** Girum Gebremeskel Kanno, Adane Tesfaye Anbesse, Mohammed Feyisso Shaka, Miheret Tesfu Legesse, Sewitemariam Desalegn Andarge

**Affiliations:** a College of Health and Medical Science, School of Public Health, Dilla University, Dilla, Ethiopia; b College of Health and Medical Science, Department of Nutrition, Dilla University, Dilla, Ethiopia; c College of Health and Medical Science, Department of Reproductive Health, Dilla University, Dilla, Ethiopia

**Keywords:** Biomass fuel, Demographic and health survey, Kitchen location, Low birth weight, Maternal report of birth size, Ethiopia

## Abstract

**Objectives:**

Household air pollution from the use of biomass fuels has been associated with low birth weight in many developing countries. This study aimed to investigate the effect of indoor air pollution from biomass fuels and kitchen location on maternal reports of child size at birth in Ethiopia.

**Study design:**

A cross-sectional study design based on the secondary data analysis was used.

**Methods:**

A secondary data analysis was conducted using data from the 2016 Ethiopian Demographic Health Survey. Birth weight from child health cards and/or mother's recall was the dependent dichotomous variable. Fuel type was classified as high-pollution fuels (i.e. wood, straw, animal dung, crop residues, kerosene, coal and charcoal) and low-pollution fuels (i.e. electricity, liquid petroleum gas, natural gas and biogas). Hierarchical logistic regression was used to assess the effect of fuel type on birth weight. Adjusted odds ratios (AORs) and their 95% confidence interval (CIs) were calculated. A p-value less than 0.05 was considered significant.

**Results:**

The prevalence of low birth weight was 17% and 26.2% among low- and high-polluting fuel users, respectively. Compared with low-polluting fuels, the use of high-polluting cooking fuels was associated with an increased likelihood of low birth weight (unadjusted crude odds ratio 1.7; 95% CI 1.3, 2.3). AOR remained at 1.7 (95% CI 1.26, 2.3) after controlling for child variables. AOR after controlling for both child and maternal factors was 1.5 (95% CI 1.1, 2.1). In the final model, the association became insignificant with an AOR of 1.3 (95% CI 0.9, 1.9). The kitchen location, gender of the baby, mother's anaemia status, maternal chat chewing and wealth index were significant factors in the final model.

**Conclusions:**

In this study, the use of biomass fuels and kitchen location were associated with reduced child size at birth. Further observational studies should investigate this association using more direct methods for measurement of exposure to smoke emitted from biomass fuels on birth weight.

## 1 Introduction

Low birth weight is defined as weight at birth <2500 g. It is a significant public health problem globally and is associated with a range of both short- and long-term consequences [1]. Low birth weight is an important marker of maternal and foetal health and nutritional status [2]. Worldwide, 15–20% of all births are reported as low birth weight, representing over 20 million births a year [3]. The great majority of low weight births occur in low- and middle-income countries, especially in the most vulnerable populations, with regional estimates of 13% in sub-Saharan Africa and 26% in Ethiopia [1,4]. In most developing countries, including Ethiopia, the data on low birth weight remain limited or unreliable, as many births occur in homes or small health facilities, and they are frequently under-reported or not reported at all in official figures, resulting in an underestimation of the prevalence of low birth weight [5].

Low weight at birth is a major predictor of prenatal mortality and morbidity. Compared with babies born at or above the low-birth-weight cut-off (2500 g), babies born with low birth weight have a higher risk of stunting, lower IQ, a higher chance of childhood death and also have increased risk for non-communicable diseases, such as diabetes and cardiovascular disease later in life [[6], [7], [8], [9]].

Numerous factors have been linked with low birth weight. A safe environment is one of the basic needs of mothers to grow a healthy baby, as well as good nutrition, rest and adequate antenatal care [10]. In addition, parasitic infections and maternal exposure to different pesticides during pregnancy have also been linked with a higher risk of low birth weight [11,12]. Several studies have also indicated that low birth weight in most developing countries has resulted from exposure to an unsafe indoor environment, mainly due to household air pollution from cooking fuels and environmental tobacco smoke (ETS) [13,14]. Combustion from these solid fuels in simple household cooking stoves contributes to household air pollution (HAP) by emitting considerably large amounts of noxious pollutants and health-damaging airborne pollutants, including particulate matter (PM), carbon monoxide (CO), nitrogen dioxide (NO_2_), formaldehyde and many other toxic polycyclic aromatic hydrocarbons (PAHs) [[15], [16], [17], [18]]. Of these noxious pollutants, carbon monoxide is a well known foeto-toxic chemical associated with poor foetal growth. Two mechanisms have been reported in the literature for this association. The first mechanism occurs when the amount of oxygen supply that must be delivered to tissues has decreased. The phenomenon, called hypoxia, occurs because carbon monoxide interacts with haemoglobin to cross the placenta, limiting the placenta's ability to transfer nutrients to the foetus. The second mechanism occurs when inhaled particulate matter from smoke impairs foetal growth by damaging cells through oxidative stress [[19], [20], [21]].

Almost 3 billion people, primarily in low- and middle-income countries, and 90% of the rural household population in developing countries still rely on high-polluting solid fuels (i.e. wood, straw, animal dung, crop residues, kerosene, coal and charcoal), burnt in inefficient, highly polluting stoves for cooking, heating and lighting, which are responsible for producing a high concentrations of particulate matter in the indoor environment [17,18,22].

In sub-Saharan Africa, the number of people using biomass fuel use has shown no significant change in three decades from 1980 to 2010, yet the population exposed to indoor air pollution has increased from 333 million to 646 million [23]. This can be translated as 76% of particulate matter air pollution worldwide occurs indoors in developing countries. When biomass fuels are burnt on traditional, typically simple, inefficient and unwanted household cooking stoves, they produce large volumes of indoor smoke or air pollutant, which exceeds the safe levels recommended by the World Health Organisation (WHO), namely a recommended 24-h mean: PM_2.5_ <25 μg/m^3^ and PM_10_ < 50 μg/m^3^) [17,18,23,24].

In the sub-Saharan Africa region, where Ethiopia is located, the leading risk factor for neonatal death (which accounts for more than half of under-five mortality) is low birth weight [25]. Global under-five mortality data indicate that interventions must be enhanced to change the current situation to achieve the sustainable development goal (SDG) target of 25 per 1000 live births by 2030 in the sub-Saharan Africa region [26].

In Ethiopia, more than 95% of households rely on biomass fuels for cooking and in almost 53% of households, food is cooked inside the house. This creates a favourable condition for indoor air pollution, which is the largest single environmental risk factor for adverse pregnancy outcomes, such as premature death and low birth weight [4,22].

Studies conducted in developing countries, such as India, Bangladesh, Pakistan and Malawi, indicate that the use of high-polluting cooking fuels was associated with low birth weight [14,[27], [28], [29], [30]]. The majority of previous studies assessing the predictors of low birth weight in Ethiopia have focused on maternal, child and sociodemographic factors only [[31], [32], [33], [34]], and maternal exposure to indoor air pollution was not taken into consideration. However, other studies [35,36] have tried to assess the impact of different fuel types on child size at birth, but their focus was localised to a small study area. Another study assessed the predictors of small birth size using the 2011 Ethiopian Demographic Health Survey (EDHS) nationwide data [37], but the impact of the type of household fuel and kitchen location on birth weight was not analysed. To fill this gap in knowledge, it is necessary to provide national empirical evidence on the magnitude of the risk posed by indoor air pollution to low birth weight in the Ethiopian context. Therefore, this study aimed to assess the relationship between maternal exposure to biomass fuel and low birth weight at the national level in Ethiopia. In addition, we tried to determine the effect of biomass fuel use and kitchen location on birth size in the 5 years preceding the 2016 EDHS.

## 2 Methods

### 2.1 Data source, setting and study design

Data for this cross-sectional study were obtained from the 2016 EDHS. The census frame is a complete list of 84,915 enumeration areas (EAs) created for the 2007 population and housing census (PHC). Two-stage stratified sampling was applied to identify eligible residential households across 645 enumeration areas (EAs). Each region was stratified into urban and rural areas, yielding 21 sampling strata. Samples of EAs were selected independently in each stratum in two stages. Implicit stratification and proportional allocation were achieved at each of the lower administrative levels by sorting the sampling frame within each sampling stratum before sample selection, according to administrative units at different levels and by using a probability proportional to size selection at the first stage of sampling. In the first stage, a total of 645 EAs (202 in urban areas and 443 in rural areas) were selected with probability proportional to EA size (based on the 2007 PHC) and with independent selection in each sampling stratum. The resulting lists of households served as a sampling frame for the selection of households in the second stage.

### 2.2 Dependent variable/outcome variable

Our dependent variable was maternal reported birth size. In Ethiopia, as in most developing countries, the majority of deliveries take place at home; therefore, information on birth weight was only obtained for small number of babies (14% of births) who were weighed at birth [4]. Information on birth weight was collected by either a written record or the mother's recall on the size of their babies. Respondents (mothers) were asked “At birth, what was the size of the baby?” The options were ‘very large’, ‘larger than average’, ‘average’, ‘smaller than average’ and ‘very small’. An infant was classified as being low birth weight (<2500 g) if the mother reported that they were ‘smaller than average’ or ‘very small’ and all other infants were labelled as not low birth weight (>2500 g).

### 2.3 Exposure variables

The main independent variables of interest for this study were cooking fuel type and kitchen location. The standard Demographic Health Survey (DHS) used an 11-fold classification of cooking fuels used in the house. The specific questions asked were “What type of fuel does your household mainly use?” and “Is the cooking usually done in the house, in a separate building, or outdoors?”. For our analysis, the main cooking fuels used was separated into two groups; namely, high-pollution cooking fuels (i.e. wood, straw, animal dung, crop residues, kerosene, coal and charcoal) and (2) low-pollution cooking fuels (i.e. electricity, liquid petroleum gas, natural gas and biogas). Kitchen location was categorised into the following three groups: (1) in the house, (2) in a separate building and (3) outdoors.

### 2.4 Other predictor variables

Other independent variables included in the model were maternal age (<20, 20–29 and 30–49 years), maternal education (none, primary, secondary or higher), maternal body mass index (BMI) [underweight, normal, overweight or obese), wealth index (1–5 from poorest to richest, calculated based on the availability of household assets using principal component analysis and provided in the dataset) [4], birth order (first, second, third, fourth or higher), gender of the child (male or female), pregnancy intention (planned, mistimed or unplanned), residence (urban or rural), chat chewing (no or yes) and alcohol drinking (no or yes).

### 2.5 Inclusion and exclusion criteria

**Fig. 1:**
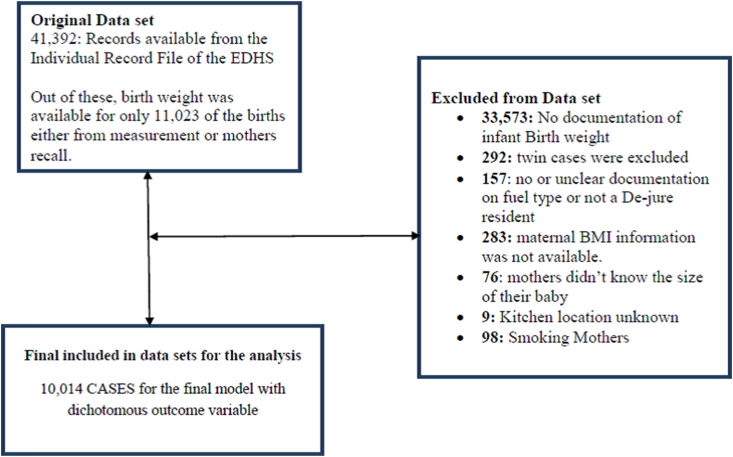
Included and excluded cases on the data management process.

A total of 44,596 births were reported during the previous 5 years and birth size data were available for 11,023 (either from the health card or mother's recall). Of these, 10,730 were singleton births and 10,014 fulfilled the inclusion criteria as shown in Fig. 1. Births with missing data for child, maternal and household factors were excluded. Mothers who smoked cigarettes were excluded from this study because they were very low in number, and all were found to be in the ‘high-polluting cooking fuel users’ category.

### 2.6 Data analyses

For the bivariate analysis of categorical variables, we used binary logistic regression. We carried out the analyses using SPSS Version 20 software. In addition to the type of fuel used, we considered other (independent) variables, including child factors (i.e. gender of the baby and birth order), maternal factors (i.e. anaemia level, BMI, age at first childbirth, chat chewing, alcohol drinking, education and pregnancy intention) and sociodemographic factors (i.e. place of residence [urban/rural], wealth index, sex of head of the household). The possibility of collinearity was checked using the Pearson's correlation matrix and the Pearson's correlation coefficients were calculated to rule out multicollinearity. Multilevel modelling was performed to adjust for cluster sampling (cluster as the primary sampling unit used in DHS). During statistical modelling, certain factors known or suspected to confound the results (such as the mother's age and BMI, parity, alcohol use, chat chewing, pregnancy intention, birth order, gender of the child, wealth index and residence) were adjusted. The Hosmer and Lemeshow test was used to check for model fitness. Adjusted odds ratios (AORs) and their 95% confidence intervals (95% CIs) were calculated. A p-value less than 0.05 was considered significant.

## 3 Results

### 3.1 Descriptive statistics

Of the 10,014 participants included in the study, child size at birth (as reported by the mother) showed 1602 (16.0%) were very small, 989 (9.9%) were smaller than average and 74.1% were average or larger than average. These results are similar to findings from the overall EDHS 2016 report, which shows 16% of births were very small, 10% were smaller than average and 73% were average or larger than average. Of the 2591 (25.9%) low birth weight infants, the majority (2538 [97.9%]) were from households using biomass fuel (such as charcoal, wood, straws or crops and animal dungs) and 1181 (45.6%) were from households where food was cooked inside the house. The dominant type of fuel used by the households with infants in Ethiopia was wood (8383 [83.7%]) and only about 3% (274) of households used electricity (Table 1).

**Table 1:** Distribution of birth sizes according to biomass fuel use, kitchen location and selected sociodemographic factors [n (%)].

Variable	Weight at birth (mother's recall)					Total (10,014)
	Very large (1796)	Larger than average (1407)	Average (4820)	Smaller than average (989)	Very small (1602)	
**Fuel types**						
Low-polluting fuels	90 (5%)	34 (2.4%)	134 (3.2%)	26 (8.4%)	27 (8.7%)	311 (3.1%)
High-polluting fuels	1706 (95%)	1373 (97.6%)	4686 (96.8%)	963 (97.4%)	1575 (98.3%)	9703 (96.9%)
**Kitchen location**						
Inside the house	738 (41.1%)	627 (44.6)	1740 (41.2%)	455 (46%)	725 (45.3%)	4286 (42.8)
In another building	887 (49.4%)	641 (45.5%)	1963 (46.5%)	436 (44.1%)	738 (46%)	4664 (46.6%)
Outdoors	171 (9.5%)	139 (9.9%)	518 (12.3%)	97 (9.8%)	139 (8.7%)	1064 (10.6%)
**Highest Education Level**						
No education	1071 (59.6%)	956 (68%)	2734 (64.8%)	705 (71.3%)	1164 (72.6%)	6631 (66.2%)
Primary	586 (32.6%)	378 (26.9%)	1169 (27.7%)	226 (22.9%)	363 (22.7%)	2722 (27.2%)
Secondary	81 (4.5%)	50 (3.6%)	223 (5.3%)	36 (3.6%)	49 (3.1%)	438 (4.4%)
Higher	58 (3.2%)	23 (1.6%)	94 (2.4%)	22 (2.2%)	26 (1.7%)	223 (2.2%)
**Place of Residence**						
Urban	228 (12.7%)	127 (9%)	480 (11.4%)	83 (8.4%)	130 (8.1%)	1048 (10.5%)
Rural	1568 (87.3%)	1280 (91%)	3740 (88.6%)	905 (91.6%)	1472 (91.9%)	8965.5 (89.5%)
**Wealth index**						
Poorest	341 (19%)	337 (23.9%)	992.5 (23.5%)	297 (30.1%)	441 (27.6%)	2408.8 (24.1%)
Poorer	415.5 (23.1%)	303 (21.6%)	950 (22.5%)	224.6 (22.7%)	407.8 (25.5%)	2301 (23%)
Middle	344 (19.1%)	321 (22.8%)	880 (20.9%)	215 (21.8%)	329 (20.6%)	2089 (20.9%)
Richer	396 (22%)	250.5 (17.8%)	774 (18.3%)	148.5 (15%)	249 (15.6%)	1818 (18.2%)
Richest	300 (16.7%)	195 (13.9%)	623.6 (14.8%)	103 (10.4%)	174 (10.9%)	1396.6 (13.9%)

The proportion of low-birth-weight infants among low-polluting fuel users was 17%, while among biomass fuel users it was 26.2%. Of the total children included in this study, 2591 (25.9%) were of low birth weight and 9703 (96.9%) infants belong to households using biomass fuels. Exposure to cooking smoke is greater when cooking takes place inside the house than in a separate building or outdoors. In this study, cooking took place in a separate building for 4664 (46.6%) households, which is similar to the EDHS report that showed 47% of households cooked food in a separate building.

### 3.2 Bivariate analysis

The bivariate logistic regression analysis revealed that there is a significant association between many potential predictor variables and child size at birth as indicated in Table 2. Infants born to mothers who live in households utilising biomass fuels were 1.7 (95% CI 1.3, 2.3) times more likely to be born with low birth weight compared with those using low-polluting fuels. Infants whose mothers reside in households where food is cooked inside the house or in a separate building were more likely to be of low birth weight than infants born to mothers from households where cooking is mostly done outdoors. Among other maternal factors, the age of the mother at first pregnancy, being moderate or mildly anaemic and low BMI were among the risk factors for having a low-birth-weight child. Similarly, female sex of the child, and children from rural areas and with a lower wealth index were at increased risk of low birth weight.

**Table 2:** Multivariable analysis of child size at birth with fuel type, kitchen location and other variables [AOR (95% CI)]^a^.

Variable	MODEL 1	MODEL 2	MODEL3
Type of fuel			
Low-pollution fuels	1	1	1
High-pollution fuels	1.7 (1.3–2.3)***	1.5 (1.1–2.1)*	1.4 (0.98–1.9)
**Kitchen location**			
Inside the house	1.3 (1.1–1.6)***	1.4 (1.2–1.7)***	1.3 (1.1–1.6)**
In another building	1.2 (1.03–1.4)*	1.3 (1.1–1.5)**	1.3 (1.15–1.5)**
Outdoors	1	1	1
**Gender of the baby**			
Male	1	1	1
Female	1.5 (1.4–1.6)***	1.5 (1.4–1.6)***	1.5 (1.4–1.7)***
**Birth order number**			
1	1		
2	0.9 (0.8–1.1)	**xxx**	
3	0.9 (0.8–1.03)	**xxx**	
4+	0.9 (0.8–1.1)	**xxx**	
**Mother's age at birth**			
<20 years		1	1
20–29 years		0.9 (0.8-.98)*	0.9 (0.8–0.97)*
30–49 years		1.1 (0.7–1.6)	1.1 (0.7–1.6)
**Maternal BMI**			
Underweight		1	
Normal		1.01 (0.9–1.1)	
Overweight		1.1 (0.8–1.4)	
Obese		0.7 (0.5–1.1)	**xxx**
**Maternal anaemia level**			
Severe		1.4 (0.95–1.9)	1.3 (0.9–1.8)
Moderate		1.3 (1.1–1.6)**	1.2 (1.03–1.5)*
Mild		1.2 (1.1–1.4)***	1.2 (1.1–1.3)*
Not anaemic		1	1
**Pregnancy intention when became pregnant**			
Planned		1	
Mistimed		1.1 (1.0–1.3)	**xxx**
Unplanned		1.02 (0.9–1.2)	**xxx**
**Maternal Chat chewing**			
No		1	1
Yes		1.2 (1.1–1.4)**	1.2 (1.1–1.4)**
**Maternal Alcohol Drinking**			
No		1	1
Yes		1.3 (1.2–1.5)***	1.3 (1.2–1.5)***
**Mother's education**			
No education		1.1 (0.8–1.6)	0.9 (0.7–1.4)
Primary		0.8 (0.6–1.2)	0.8 (0.5–1.1)
Secondary		0.7 (0.5–1.1)	0.8 (0.5–1.1)
Higher		1	1
**Type of residence**			
Urban			1
Rural			1.0 (0.8–1.2)
**Wealth index**			
Poorest			1.5 (1.2–1.9)***
Poorer			1.4 (1.1–1.7)**
Middle			1.3 (1.03–1.6)*
Richer			1.1 (0.9–1.3)
Richest			1
**Sex of head of the household**			
Male			1
Female			1.1 (0.96–1.3)

AOR, adjusted odds ratio; BMI, body mass index; CI, confidence interval.

*p < 0.05, **p < 0.01, ***p < 0.001.

a Model 1: association of cooking fuel and kitchen location on low birth weight was assessed with child variables; Model 2: model 1 plus addition of maternal variables; Model 3: model 2 plus demographic variables.

### 3.3 Multivariable analysis

We have analysed the effect of fuel type and kitchen location on maternal report of child size at birth, hierarchically. In the initial stage (model 1) when the association of cooking fuel and kitchen location was assessed with child factors, the use of biomass fuel had a significant effect on maternal report of child size at birth. Apart from the type of fuel used, the kitchen location and being a female child also had a significant effect on maternal reported birth size (Table 2).

In the second stage (model 2), the selected variables from model 1 were added to the maternal characteristics, such as the age of the mother at first birth, maternal educational status, BMI, anaemia level, maternal chat chewing and maternal alcohol drinking. There was a reduction in the strength of the effect of fuel type on low birth weight, but the association remained significant. Other maternal factors that significantly impacted the child's birth size included maternal anaemia, maternal chat chewing and maternal alcohol drinking. Being a female infant remained a significant effect on low birth weight in model 2 and its effect size was unchanged at this stage.

In the final model (model 3), child, maternal and demographic variables were included with the exposure variables to examine the effect on low birth weight. At this stage, mothers who live in households using biomass fuels tend to have a higher likelihood of having a low-birth-weight infant, but the association was insignificant (AOR 1.4; 95% CI 0.98, 1.9); however, kitchen location remained a significant predictor of low birth weight. Cooking in the house (AOR 1.3; 95% CI 1.1, 1.6) or in a separate building (AOR 1.3; 95% CI 1.2, 1.5) were found to have an almost similar significant effect on birth size compared with cooking food outdoors. Being a female child was also a significant predictor in model 3; its strength was found to be similar and strong in all three models. Maternal age, anaemia level, pregnancy intention, chat chewing, alcohol drinking and wealth index were also found to be significant predictors of child size at birth in model 3 (Table 2).

## 4 Discussion

According to the descriptive findings of the study, one-quarter of children were born with low birth weight; approximately 62% of these children were ‘very small’ and 38% were ‘smaller than average’. This finding is comparable with results from rural India, where the National Family Health Survey [38] found the level of low birth weight to be 23%, and another finding from India that identified 23.8% in Dehradun [39] and 23% in Rural Karnataka [40]. This finding is also consistent with a meta-analysis done in subs-Saharan Africa where, indoor air pollution from biomass fuel use was linked with low birthweight [48].

In Ethiopia, only 3% of the households use low-polluting fuels and the main source of fuel for most households (about 85%) is wood. This level of highly polluting cooking fuels among households in Ethiopia is higher than findings from India (72.9%) [28], Malawi (80%) [30] and Ghana (66%) [41].

Regarding the association between birth weight and fuel type, children of mothers from households with high-polluting fuels were about two times more likely to have low birth weight on bivariate analysis and intermediate models. However, the use of biomass fuel was not significantly associated with birth weight after all the confounding variables were controlled on multivariate analysis in the final model. A similar finding was revealed in a study by the WHO on indoor air pollution from biomass fuel use and the risk of low birth weight [42] and a study from Ghana [41]. Another study from Malawi using DHS data revealed an increased, but insignificant, association between biomass fuel use and low birth weight [30]. Similarly, a study conducted using Indian DHS data, identified a higher risk of low birth weight from biomass fuel use at bivariate analysis, but an insignificant relationship after adjustment for other predictor variables [28].

However, other findings from different studies showed that using biomass fuels was associated with a higher risk of low birth weight; using biomass fuel was associated with a two-fold increased risk on low birth weight in Lanzhou, China [43], and a 175 g reduction in mean birth weight in children born to mothers that used biomass fuels in Zimbabwe[14]. A similar finding was reported using the Pakistan DHS data, where children born in households with biomass fuel (wood) users were found to be 41% more likely to have low birth weight than children born in households using cleaner fuel types, such as natural gas [29]. According to this study finding, the wealth index was found to be a significant predictor of low birth weight. Infants from poorer households were found to be at higher risk of being low birth weight than infants from richer households. The strength of the association between wealth index and birth size decreases from the poorest to the richest population groups, which could be a clear indication that most solid biomass fuels are either cheap or free (in the case of agricultural residues and animal dung) compared with low-polluting fuel types, such as electricity, liquid petroleum gas, natural gas and biogas, which are relatively expensive.

The current study identified that cooking either inside the house or in a separate building was associated with the maternal reporting of smaller child size at birth compared with cooking outdoors. Cooking food in the house was also shown to be associated with low birth weight in a study conducted in the Wolaita zone, southern Ethiopia [35], and the Dang district of Nepal [44], where Children born to mothers who regularly cook inside the house more frequently reported a low-birth-weight baby than mothers who cooked outdoors. This might be due to the risks associated with the type of fuel use, exposure time, ventilation status and the efficiency of the cooking stove. The WHO set a public health standard for indoor air pollutants (24-h mean: PM_2.5_ <25 μg/m^3^ and PM_10_ < 50 μg/m^3^), which can be attained through the use of cleaner fuels, well-ventilated households and kitchens, efficient cooking stoves and reduced exposure times. In most developing countries, including Ethiopia, there is limited access to improvements in this area, thus biomass fuel use together with longer exposure times, poor ventilation and unimproved cooking stoves increases the likelihood of maternal exposure to a higher level of pollutants, which, in turn, results in low birth weight [17,18,23,24].

According to our findings, cooking inside the house and cooking in a separate building has an almost similar effect on birth size when compared with cooking outdoors. This is a clear indication that, unless efficient stoves are in place with adequate ventilation when using solid biomass fuels, the effect of cooking inside the house or in a separate kitchen might not have a difference. This is apparent in countries such as Ethiopia, where widespread use of improved cooking stoves has not taken place for various reasons, such as low incomes, lack of infrastructure, slow market penetration into remote villages, lack utilisation knowledge and information gaps [45].

Our findings were contrary to similar research conducted in Bangladesh [27], where the cooking place was not significantly associated with child size at birth. One reason for the difference might be due to the difference in the kitchen location between the two countries (82.4% of Bangladesh residents cook outdoors, while this is only 9.5% in Ethiopia). Since the outdoor environment is more ventilated than the indoor environment, outdoor cooking is expected to reduced exposure to noxious pollutants emitted from biomass fuel combustion compared with indoor cooking.

Ethiopian female infants were more likely to be of low birth weight than their male counterparts. This finding was similar to results from other studies, including research from Japan [46] and Northern Ethiopia [33]. In addition, infants of anaemic women had a marginally higher possibility of being low birth weight than infants of non-anaemic women. From a study conducted in Northern Ethiopia, anaemic women were found to be nine times more likely to deliver an infant with low birth weight [33] and a similar result was found from the national Family Health Survey-IV in India [28]. Similarly, a marginal association was found between maternal chat chewing and birth weight, which is consistent with findings from a study conducted in Yemen [47].

### 4.1 Methodological strengths and limitations

The strength of this study is that the findings can be generalised at the country level since the study utilised data from a nationally representative household survey. However, children were classified as low birth weight or not low birth weight based on the mother's subjective judgment, which might introduce measurement bias on the outcome ‘low birth weight’. This could have implications for the results. In addition to the reliance on self-report of birth size, the cross-sectional design might pose problems in establishing the temporal link between exposure and outcome. This study assumed that maternal exposure to biomass fuels was a phenomenon that occurred repeatedly over a long time before pregnancy, which might not always be the case.

### 4.2 Conclusion

The use of biomass fuel and kitchen location was associated with child size at birth. Our findings have important programme and policy implications for countries such as Ethiopia, where large proportions of the population rely on high-polluting biomass fuels for cooking. Furthermore, future studies should investigate the association using more direct methods for measurement of exposure to smoke emitted from biomass fuels on birth weight.

## Ethical approval

This secondary analysis was exempted from ethical review approval because it used publicly available, de-identified data. However, a request to access datasets from the DHS was made, and a letter of permission to use the data set was obtained before the analysis was conducted.

## Funding

The authors have no support or funding to report.

## Data availability statement

The data are available from the Demographic and Health Survey programme website. These data are publicly available online and can be accessed at the following website by selecting the specific country, Ethiopia. http://dhsprogram.com/data/available-datasets.cfm.

## Authors’ contributions

Conceptualisation: Girum Gebremeskel Kanno; Formal analysis: Girum Gebremeskel Kanno and Sewitemariam Desalegn Andarge; Methodology: Girum Gebremeskel Kanno and Sewitemariam Desalegn Andarge; Project administration: Girum Gebremeskel Kanno; Supervision: Adane Tesfaye Anbesse, Mohamed Feyisso Shaka and Miheret Tesfu Legesse; Writing – original draft: Girum Gebremeskel Kanno and Mohamed Feyisso Shaka; Writing – review & editing: Girum Gebremeskel Kanno, Mohamed Feyisso Shaka and Adane Tesfaye Anbesse, Miheret Tesfu Legesse.

## Declaration of competing interest

The authors declare that they have no known competing financial interests or personal relationships that could have appeared to influence the work reported in this paper.
